# Thermo-Magneto-Electric Generator Arrays for Active Heat Recovery System

**DOI:** 10.1038/srep41383

**Published:** 2017-02-01

**Authors:** Jinsung Chun, Hyun-Cheol Song, Min-Gyu Kang, Han Byul Kang, Ravi Anant Kishore, Shashank Priya

**Affiliations:** 1 Center for Energy Harvesting Materials and System (CEHMS), Bio-Inspired Materials and Devices Laboratory (BMDL), Virginia Tech, VA 24060, USA

## Abstract

Continued emphasis on development of thermal cooling systems is being placed that can cycle low grade heat. Examples include solar powered unmanned aerial vehicles (UAVs) and data storage servers. The power efficiency of solar module degrades at elevated temperature, thereby, necessitating the need for heat extraction system. Similarly, data centres in wireless computing system are facing increasing efficiency challenges due to high power consumption associated with managing the waste heat. We provide breakthrough in addressing these problems by developing thermo-magneto-electric generator (TMEG) arrays, composed of soft magnet and piezoelectric polyvinylidene difluoride (PVDF) cantilever. TMEG can serve dual role of extracting the waste heat and converting it into useable electricity. Near room temperature second-order magnetic phase transition in soft magnetic material, gadolinium, was employed to obtain mechanical vibrations on the PVDF cantilever under small thermal gradient. TMEGs were shown to achieve high vibration frequency at small temperature gradients, thereby, demonstrating effective heat transfer.

Small unmanned aerial vehicles (UAVs) (less than 1 kg) are continuously being explored for surveillance and security^1^,^2^,^3^,^4^,^5^,^6^. Recent focus in UAV design has been on integration of solar modules with wingspan in order to increase the endurance^7^,^8^,^9^,^10^,^11^. However, in order to generate sufficient power for extended flight time through solar cell modules, large surface area is required to maximize the output power and dissipate heat arising during cyclic operation^12^,^13^,^14^,^15^. Conventional solar modules exhibit 0.4% decrease in output power per 1 °C rise in temperature beyond the threshold operating temperature^16^. The temperature on the back-side of module in UAV’s can rise up to 70 °C^17^. Similarly, there is increasing emphasis on development of the heat management system for data centers. The large packing density of transistors with increasing processing speed leads to wasted heat, thereby, necessitating the deployment of heat dissipation system for safe operation. Rising central processing unit (CPU) temperature results in shortening of life time, malfunction, and failure^18^,^19^,^20^. This failure rate of CPU tends to increase exponentially with rising temperature.

The thermo-magneto-electric energy harvesting is promising methodology for high efficiency low grade heat management. The thermo-magneto-electric generator (TMEG), composed of soft and hard ferromagnetic materials (here we used Gd and Nd as soft and hard magnets respectively), absorbs generated heat from the source and converts it into useful electrical energy with residual heat dissipated into the sink^21^. The driving force for TMEG relies on thermally induced second order ferromagnetic to paramagnetic phase transition under thermal gradient in soft magnet^22^,^23^,^24^,^25^,^26^,^27^. This phase transition creates mechanical vibration between hard magnet attached to the heat source (hot-side) that is always above the Curie temperature (294 K) and soft magnet attached on the spring in contact with the sink (cold-side). The soft magnet oscillates between the hot-side and cold-side undergoing ferromagnetic to paramagnetic phase transition (spring moving upward) and paramagnetic to ferromagnetic phase transition (spring moving downward), and this mechanical energy is converted into electrical energy through piezoelectric material. The thermal energy from the heat source is transferred and dissipated to heat sink through the soft ferromagnetic material.

Since the prediction of TMEG in 2007, mostly theoretical work has been conducted in literature in optimizing the output power density and conversion efficiency^21^. Recently, carbon nanotube thermal interface has been employed for increasing the heat transfer from soft ferromagnetic material^28^. Theoretical approach towards optimizing the interactions between magnets and magnitude of device-relevant parameters that influence the dynamic behavior of the overall system has been reported^29^. Here, we demonstrate feasible and practical TMEG arrays, composed of flexible and lightweight piezoelectric polyvinylidene difluoride (PVDF) bimorph cantilevers. Under thermal gradient, the soft magnet (Gd) motion results in mechanical vibration of the PVDF cantilevers, thereby, generating electricity. This novel design of TMEG arrays achieves vibration frequency from 1 to 3 Hz and generates 17 V and 158 μW under the thermal gradient of 80 °C. TMEG arrays were attached to a central processing unit (CPU) inside the desktop to confirm the dual role of thermal energy harvesting and heat dissipation.

## Results and Discussion

**Figure 1:**
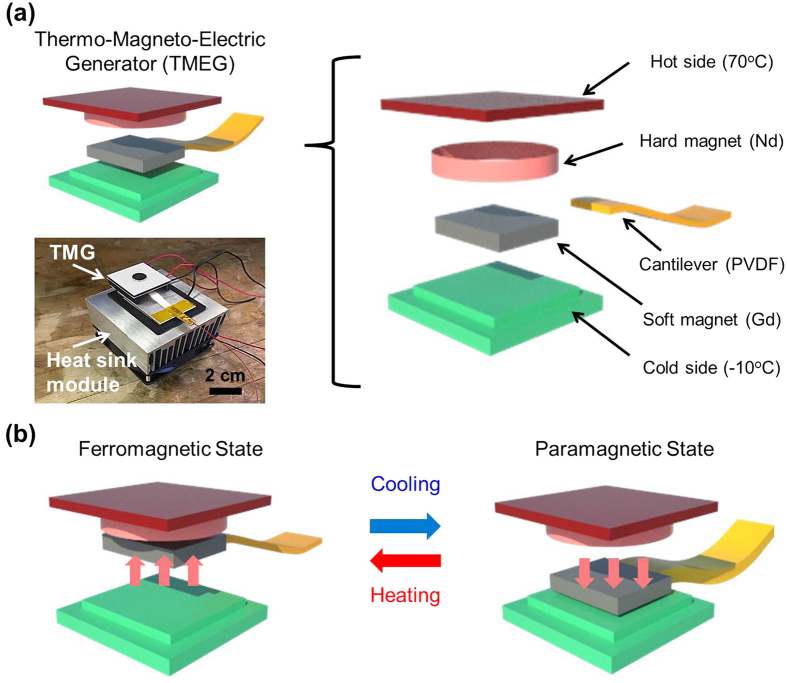
Fabrication process of TMEG. (**a**) Schematic diagram of TMEG. The picture shows fabricated device image with heat sink module. (**b**) Schematic representation for TMEG operating through second order phase transition occurring in soft magnet during periodic cooling and heating.

The schematic representation of the fabrication process for TMEG is shown in Fig. 1a and detailed information is described in the experimental section. The generator comprises of three parts: hot-side with Peltier heater (4 × 4 cm^2^) to which disk-type hard magnet (Nd) with 1 cm diameter was attached, the unimorph piezoelectric PVDF cantilever with soft ferromagnetic material (Gd, 4 × 8 mm^2^), and cold-side with Peltier cooler. The inset shows the optical image of fabricated TMEG with total size of 10 × 10 cm^2^ and aluminum heat sink. Cooling fan is mounted underneath the cold-side for improving the heat transfer through forced convection. In the initial state, Gd attached to the PVDF cantilever, is attracted to the hard magnet on the hot-side due to magnetic force of attraction. As Gd makes contact with Nd, it loses attraction force due to phase transition and the cantilever is pulled back towards the cold-side by restoring force of cantilever. After making contact with the cold-side, Gd is cooled below the Curie temperature and it again restores its ferromagnetic state which results in rise of magnetic force of attraction with the hard magnet, as shown in Fig. 1b. This periodic cycle continues to create mechanical deformation in the unimorph PVDF cantilever, and the cantilever generates electricity due to direct piezoelectric effect and moves the thermal energy from hot-side to cold-side repeatedly.

**Figure 2:**
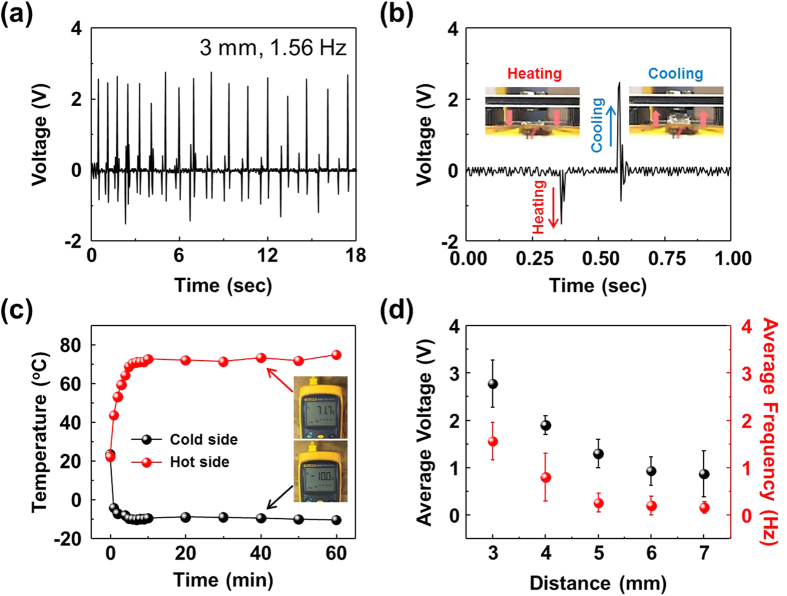
Electrical output performance of TMEG. (**a**) Output voltage of unimorph cantilever based TMEG under thermal gradient of 80 °C. (**b**) Extended output voltage during heating and cooling. The insets show snapshots of positions of soft ferromagnetic material. (**c**) Measured temperatures on the surfaces of cold-side and hot-side. (**d**) Average output voltage and vibration frequency of TMEG as a function of distance between hard magnet and cold-side.

TMEG operation was characterized under ambient condition similar to that for the UAVs at altitudes of ~60,000 ft where the outside temperature decreases up to ~−80 °C^29^. Figure 2a shows output voltage of the fabricated TMEG with unimorph PVDF cantilever at a thermal gradient of 80 °C. It was found to generate maximum output voltage of 2.6 V under vibration frequency of 1.56 Hz with travel distance of 3 mm between hard magnet and cold-side. The output voltage of the unimorph cantilever based TMEG shows different polarity depending upon the bending direction of PVDF cantilever, as shown in the inset of Fig. 2b. To confirm the polarity of generated voltage with different connection, TMEG is connected with forward and reverse connection to the oscilloscope. The polarity is symmetrically changed under different connection (see Supplementary Fig. S1). This result indicates that the measured voltage is generated from the piezoelectric PVDF through bending motion. To apply a constant thermal gradient, surface temperatures of the hard magnet and cold-side were maintained and measured for 1 h. The thermal gradient (*∆T*) was saturated to 80 °C (hard magnet at 70 °C and cold-side at −10 °C) after 10 min, as shown in Fig. 2c. Surface temperatures on the hard magnet and hot-side were not significantly different, which indicates that heat was effectively transferred from the Peltier heater to hard magnet. (see Supplementary Fig. S2).

For continuous operation of TMEG, specific distance between hard magnet and cold-side is required, which not only maintains the thermal gradient but also provides space for deformation of the PVDF cantilever. Figure 2d shows average output voltage and vibration frequency of the unimorph cantilever based TMEG as a function of gap distance. It can be clearly observed that small gap between hard magnet and cold-side increases output voltage and vibration frequency up to 2.6 V and 1.56 Hz, compared with large gaps. This indicates that there exists optimum separation distance between the magnets for a given thermal mass. TMEGs with less than 3 mm gap distance did not provide measurable electrical response due to very small displacement of piezoelectric material. Small distance between hard magnet and cold-side results in increase of vibration frequency due to decrease of the travel time of soft ferromagnetic material and restoring force of cantilever. The output power (*P*_out_) can be defined as: *P*_out_ = *VI* = *Efη*, where *E* is the mechanical energy applied on piezoelectric material, *f* is the bending frequency, and *η* is the energy conversion efficiency. Thus, high output voltage could be generated by increasing vibration frequency (*V* = *Efη*/*I*). The mechanical energy (*E*) can be defined as *E* = ∫*Fdx* = ∫*c*/(*x* + *t*)^2^*dx*, where *F* is the magnetic force of soft ferromagnetic material, *x* is distance between hard magnet and cold-side, *c* represents material characteristics, and *t* is a shape related constant^21^,^30^. Thus, this enhanced output power is attributed to the vibration frequency^31^,^32^,^33^ and magnetic force which increases the mechanical energy applied on to piezoelectric material.

**Figure 3:**
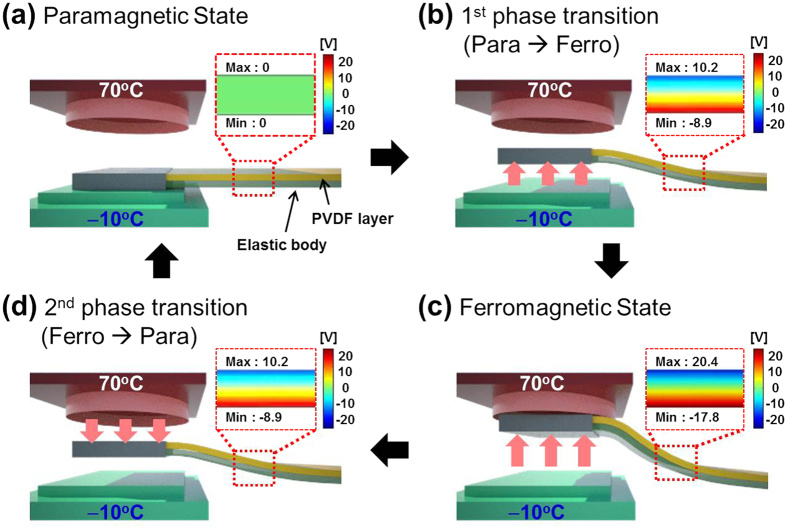
Working mechanism and numerical analysis of TMEG. (**a**) Paramagnetic state, (**b**) first phase transition during cooling, (**c**) ferromagnetic state, and (**d**) the second phase transition during heating. The insets show piezoelectric potential distributions of PVDF cantilever calculated by COMSOL Multiphysics software.

Next, the coupling of thermal gradient induced phase transition and piezoelectric effect generated by vertical oscillation of soft ferromagnetic material were systematically investigated, as shown in Fig. 3. To achieve high strain in PVDF layer, the PET elastic body was attached at bottom of PVDF according to the neutral axis theory^31^. The stress distribution significantly depends on the position of the neutral axis, which is determined from the condition that the resultant axial force acting on the cross-section is zero. Additionally, we calculated the piezopotential distributions inside the cantilever along the vertical direction by using a simple rectangular model composed of 200 μm-thickness PVDF film on the PET under the load of 640 μN. The material parameters of the PVDF, taken from the COMSOL simulation software, were used for the finite element analysis. The piezoelectric coefficient (*d*_31_) of PVDF is −32.5 pC/N and PVDF was assumed to be a dielectric polymer. The piezoelectric potential of a fully coupled electro-mechanical system was calculated from the following piezoelectric coupled equations:^34^,^35^


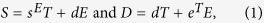


where *S* is strain, *s* is compliance, *T* is applied stress, *D* is the electric charge density displacement, *ε* is permittivity, and *E* is electric field strength. In paramagnetic state, (a) there is no strain in piezoelectric, and thus no piezoelectric potential is generated. By cooling soft ferromagnetic material below Curie temperature, the phase transition from paramagnetic to ferromagnetic phase occurs. In ferromagnetic phase, soft magnetic material is attracted to hard magnet by magnetic force, and bending strain is applied to piezoelectric cantilever, resulting in piezoelectric potential. (b) Maximum calculated potential difference of 38.2 V was generated as soft and hard magnets made contact. (c) After making contact with hard magnet, the temperature of soft magnet is increased above the Curie temperature. This results in second order magnetic phase transition from ferromagnetic to paramagnetic phase, resulting in cantilever movement towards the cold-side. (d) This cycle is continuously repeated under thermal gradient. (see Supplementary Fig. S3 and Supplementary movie 1) To confirm the magnetic characteristics of soft ferromagnetic material (Gd), the temperature-dependent magnetization of Gd was measured after field-cooling at 500 Oe, which provides transition temperatures. (see Supplementary Fig. S4).

**Figure 4:**
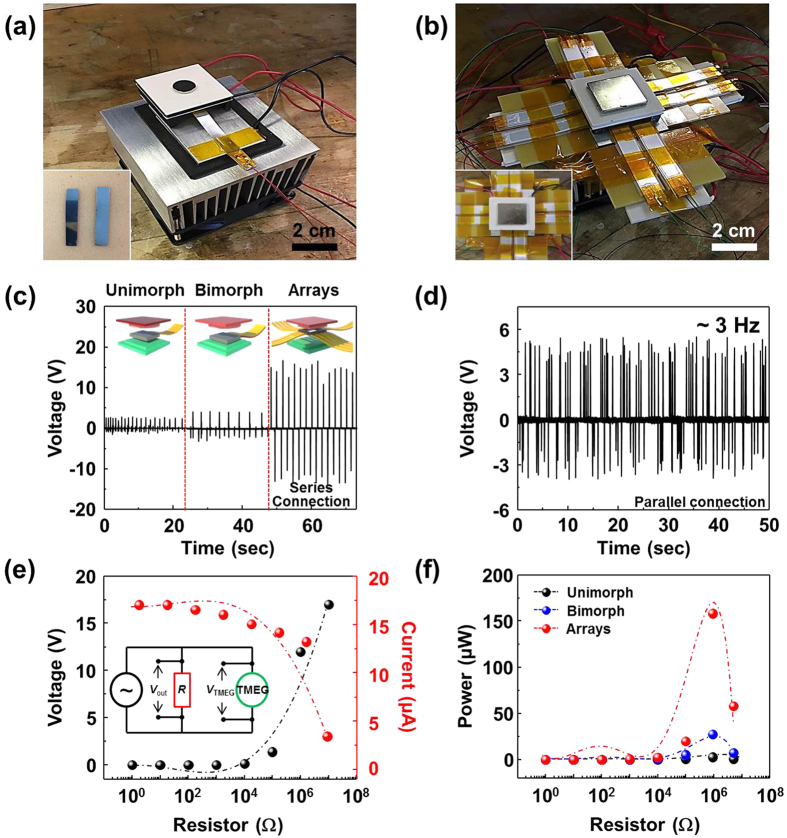
Electrical outputs and power of TMEGs. Optical images of fabricated (**a**) bimorph cantilever based TMEG and (**b**) TMEG arrays composed of bimorph cantilevers. (**c**) Output voltages of unimorph and bimorph cantilever based TMEG, and arrays in series connection. (**d**) Output voltage and vibration frequency of TMEG arrays in parallel connection. (**e**) The output voltage and current, and (**f**) the output power of the unimorph and bimorph cantilever based TMEG, and arrays with the resistance of external loads from 1 Ω to 10 MΩ.

To obtain high output from TMEG, bimorph cantilever based TMEGs were fabricated by stacking two PVDF with opposite poling direction and TMEG arrays composed of eight bimorph cantilever were prepared as shown in Fig. 4a and b. Compared with unimorph cantilever based TMEG, the output voltage of bimorph cantilever based TMEGs is enhanced up to 4.2 V. Using series connected eight bimorph cantilever arrays, the TMEG shows maximum output voltage of 17 V, which is 6 times higher than that of unimorph cantilever based TMEG. This indicates that high output voltage from TMEG can be achieved through bimorph and arraying process. To control vibration frequency of TMEG, eight bimorph cantilevers of TMEG arrays are connected in parallel, resulting in an enhanced vibration frequency of 3 Hz, compared with unimorph cantilever based TMEG. To optimize the output power of TMEG, the output voltage of TMEG was measured with external loads varying from 1 Ω to 10 MΩ, as shown in Fig. 4e. The output voltage significantly increases with increasing resistance, while the output current decreases. The electrical output of unimorph, bimorph, and arrays of TMEG at variable external loads were measured by oscilloscope with internal impedance of 10 MΩ, as shown in the inset of Fig. 4e. Here, *V*_out_ is the voltage drop across resistance *R*, which can be measured with an oscilloscope. The current (*I*_out_) through the resistance R could be calculated as *V*_out_/*R*, (see Supplementary Fig. S5) and the output power (*P*_out_) of TMEG could be defined as *V*_out_*I*_out_ with various resistances. It should be noted that *R* is required to be less than 10 MΩ due to the resistance of oscilloscope^36^. Since the oscilloscope (internal resistance 10 MΩ) and external load are in parallel, the equivalent resistance of the circuit would be slightly lower than the external load and therefore the actual power output of TMEG would be slightly higher than the reported value. Instantaneous power of 158 μW across the resistance of 0.91 MΩ was obtained, as shown in Fig. 4f, giving over 64-fold power enhancement, compared to the unimorph cantilever based TMEG.

**Figure 5:**
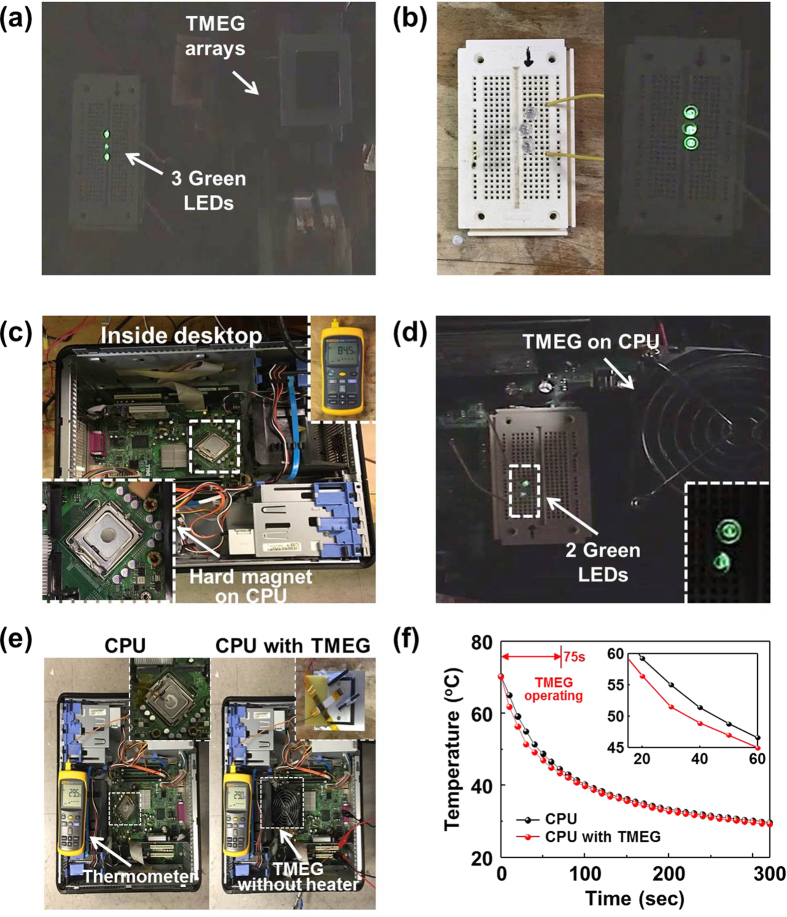
Demonstrations of the TMEG as a sustainable power source and heat recovery system. (**a**) Photograph and (**b**) enlarged image of three commercial green LEDs driven by TMEG arrays. (**c**) Photograph of a hard magnet attached to the CPU inside desktop and measured temperature on the CPU. (**d**) Photograph of two commercial green LEDs driven by bimorph cantilever based TMEG. (**e**) Snapshots of TMEG attached to CPU inside desktop and (**f**) surface temperatures on CPU with TMEG and without TMEG.

To demonstrate the capability of TMEG in dual mode operation, as power source and heat dissipator, we implemented two sets of practical applications. First, TMEG was directly connected to three green commercial LEDs with rectifier unit. All of series connected LEDs were simultaneously powered by TMEG arrays, as shown in Fig. 5a and b. Second, the bimorph cantilever based TMEG without Peltier heater was mounted onto a central processing unit (CPU) inside the desktop (Fig. 5c and d). Cooling system for the CPU is required to dissipate heat raised during operation. When operating desktop, the measured temperature on the surface of the CPU was ~84.5 °C. It can be clearly seen that TMEG successfully harvests thermal energy from the desktop and generates sufficient electrical power for two commercial green LED bulbs (see Supplementary movies 2 and 3). For determining the heat dissipation capabilities, as shown in Fig. 5e, TMEG (without heater) was attached to CPU inside desktop and surface temperature of CPU was measured after turning on the power and turning off the cooler. The figure clearly shows that CPU cooling was accomplished with TMEG which operated for 75 s under residual thermal gradient. The CPU shows lower surface temperature (>3 °C) as shown in Fig. 5f. Using the same measurement method, surface temperatures on Peltier heater were also measured, and the results showed a lower surface temperature (>2 °C) with the operation of TMEG at 89 s (see Supplementary Fig. S6). Assuming a lumped mass system, the governing equation for a cooling object in the atmosphere can be expressed by using following expression:


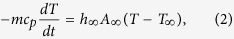


where *t* denotes time, *T* = *T* (t) is the temperature, *m* is collective mass, *c*_*p*_ is the effective specific heat capacity, *A*_∞_ is the net exposed area of the cooling object, *h*_∞_ represents cooling coefficient in air and *T*_∞_ is the ambient temperature. The TMEG essentially increases the cooling rate by adding an additional term to the right side of equation (2):


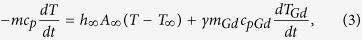


where *m*_*Gd*_ and *c*_*pGd*_ are the mass and specific heat capacity of the soft magnet respectively, and *γ* denotes a factor, which is unity when soft magnet is in contact with the cooling object and zero otherwise. As shown in the Supplementary Fig. S7, we estimate that a TMEG using one soft magnet produces cooling rate of 1 °C per minute faster than the normal dissipation. The effect increases almost linearly with increase in the number of soft magnets. These results show the feasibility of TMEG as a power source and as heat dissipation device.

## Conclusion

In summary, we report novel TMEG arrays, composed of flexible and lightweight polyvinylidene difluoride (PVDF) bimorph cantilevers, for active heat dissipation in electronic devices and solar modules. Under thermal gradient, the ferromagnetic phase transition of soft magnet (Gd) generates mechanical vibrations, resulting in piezoelectric output power generation. This design of TMEG arrays achieved the vibration frequency from 1 to 3 Hz in parallel connection and electrical outputs of 17 V and 158 μW in series connection. Since the ambient temperature at altitudes of ~60,000 ft decreases up to ~−80 °C, larger thermal gradient (∆*T* > 80 °C)^29^ and reliable output generation could be expected than that reported in this study. Thus, reliable output generation could be expected than that reported in this study. Thus, expected thermal gradient for solar-powered UAV operation is more than 150 °C. TMEG arrays attached to a central processing unit (CPU) inside the desktop showed feasibility of thermal energy harvesting from wasted heat source.

## Methods

### Fabrication of unimorph PVDF cantilever based thermo-magnetic generator (TMEG)

A gadolinium (Gd, 99.9% purity) foil of 127 μm thickness (Alfa Aesar Co., Inc., USA) was used to prepare soft ferromagnetic materials for the fabrication of thermo-magnetic generator, as described previously. Briefly, prior to locating Gd foil between the hot-side and cold-side of the device, neodymium (Nd) of 1.58 mm thickness (MAGCRAFT, USA) with a sufficiently high Curie temperature than that of Gd was attached to Peltier heater using polysynthetic silver thermal paste to enhance the heat transfer from hot-side to hard magnet, and dried for 1 h on hot plate at 60 °C. The Peltier cooler was mounted onto an aluminum heat sink with fan for past cooling up to −10 °C. To control distance between hard magnet and cold-side, the spacers were made from an insulating plastic film with thickness ranging between 3 mm to 7 mm by using CubePro 3D printer (3D Systems, Inc., USA). A soft ferromagnetic material was suspended on unimorph cantilever-type PVDF of 200 μm thickness with vertical Pt electrodes (KUREHA, USA), and then located adjacent to cold-side for generating restoring force like a spring.

### Fabrication of TMEG arrays

A large Nd magnet of an area of 4 × 4 cm^2^ was used for hard magnet and attached to the Peltier heater. Bimorph PVDF cantilevers were fabricated by stacking of two layers with opposite poling direction. The bimorph cantilevers were located on each edge of hard magnet. In order to increase output voltage and current of TMEG, the bimorph cantilevers were connected in series and parallel.

### Measurement of electrical output and characteristics

To detect voltages generated by TMEG, KEYSIGHT DSO 1014 A Oscilloscope was used for electrical measurements. The resistance of the oscilloscope is 10 MΩ. The magnetic properties of the soft ferromagnetic material were characterized by an Evercool physical property measurement system (PPMS, Quantum Design).

## Additional Information

**How to cite this article:** Chun, J. *et al*. Thermo-Magneto-Electric Generator Arrays for Active Heat Recovery System. *Sci. Rep.* **7**, 41383; doi: 10.1038/srep41383 (2017).

**Publisher's note:** Springer Nature remains neutral with regard to jurisdictional claims in published maps and institutional affiliations.

## Supplementary Material

Supplementary Information

Supplementary Movie 1

Supplementary Movie 2

Supplementary Movie 3
