# Supplementary material for: Thermo-Magneto-Electric Generator Arrays for Active Heat Recovery System
Source: Sci Rep. 2017 Feb 1;7:41383. doi: 10.1038/srep41383 (PMC5286423; doi:10.1038/srep41383)
Supplement: Supplementary Information [file srep41383-s1.pdf]

Supplementary Information

Thermo-Magneto-Electric Generator Arrays for Active Heat Recovery System

Jinsung Chun[1], Hyun-Cheol Song[1], Min-Gyu Kang[1], Han-Byul Kang[1], Ravi Anant Kishore[1] and Shashank Priya[1]*

[1]*Center for Energy Harvesting Materials and System (CEHMS), Bio-Inspired Materials and Devices Laboratory (BMDL), Virginia Tech, VA 24060, USA.*

Supplementary Figures

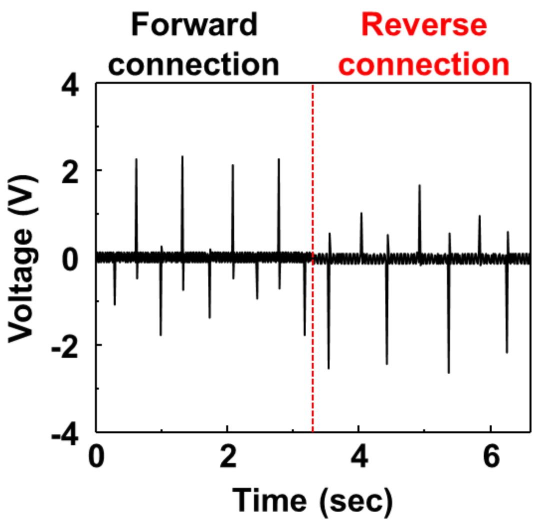

Figure S1. Output voltage signal from single cantilever based TMEG measured in the forward and reverse connection.

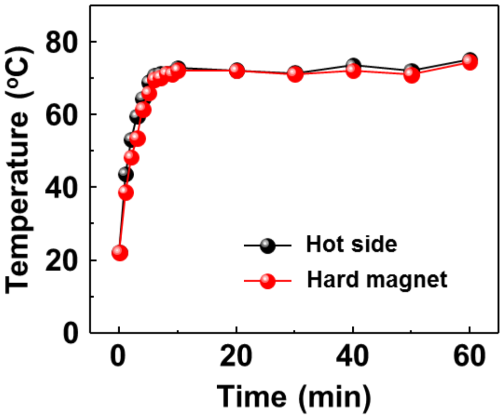

Figure S2. Measured temperature on the surfaces of hot-side and hard magnet in TMEG.

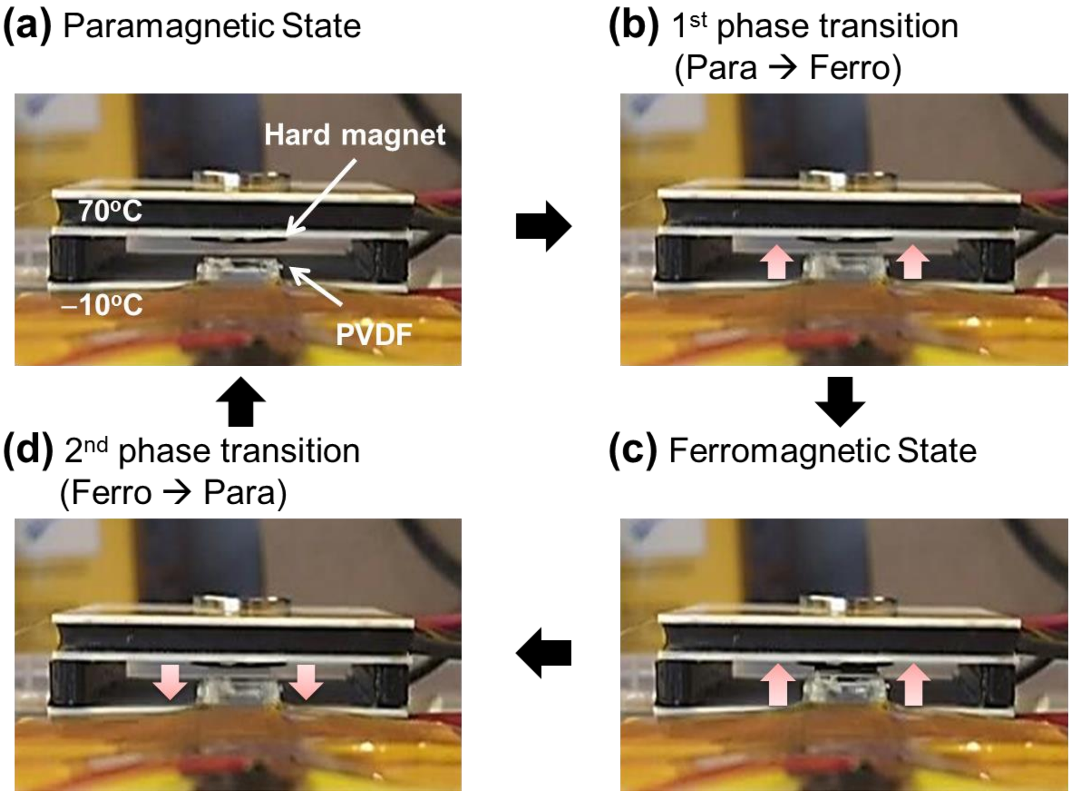

Figure S3. Snapshots of working mechanism of unimorph cantilever based TMEG. (a) paramagnetic state, (b) the first phase transition during cooling, (c) ferromagnetic state, and (c) the second phase transition during heating.

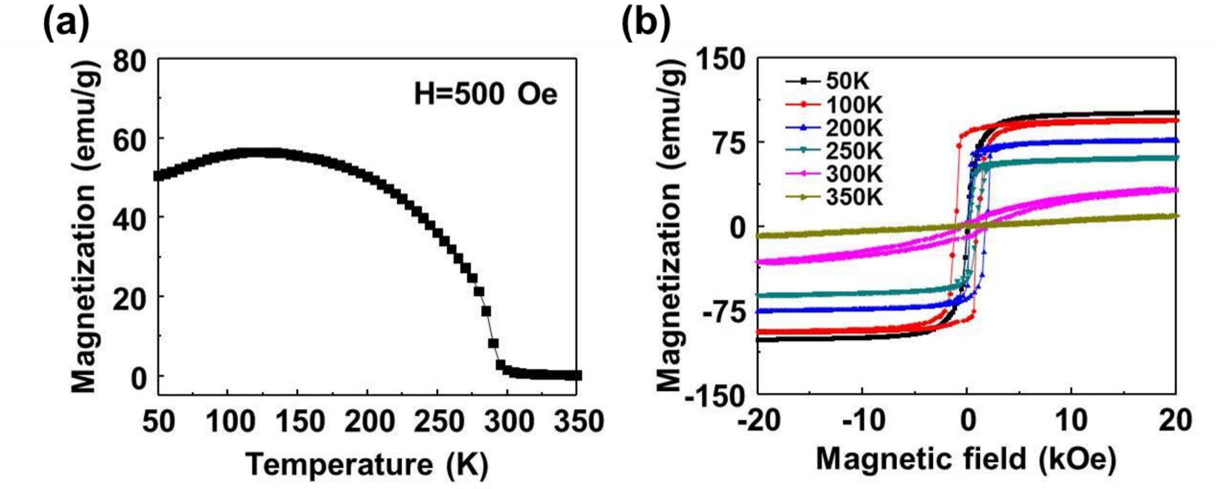

Figure S4. Magnetic characteristics of soft ferromagnetic material (Gd) (a) The temperature-dependent magnetization measured after field-cooling at 500 Oe and (b) the magnetization-magnetic field hysteresis curves.

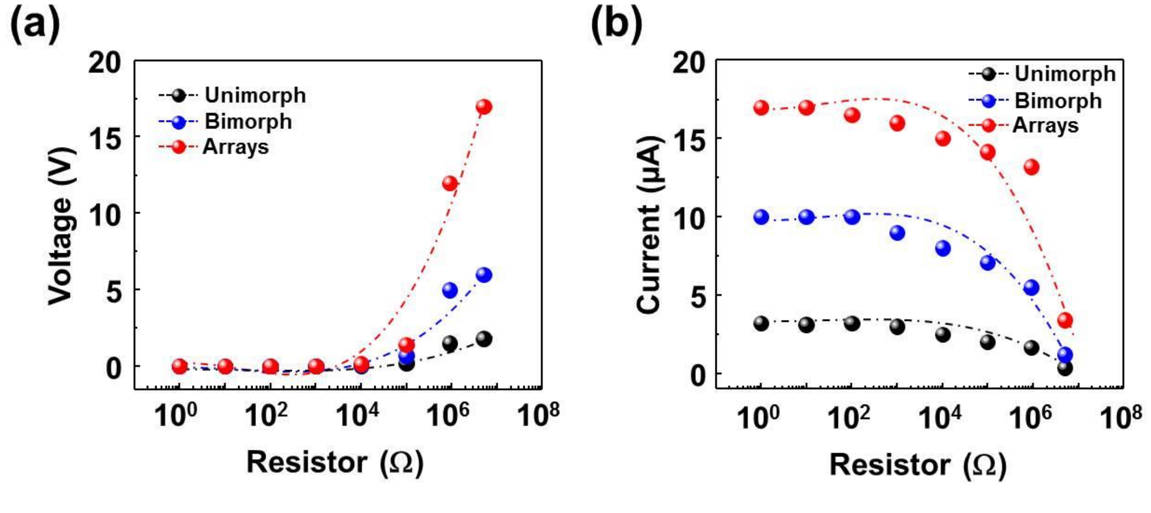

Figure S5. Electrical output performance of TMEGs. (a) The output voltage and (b) current of the unimorph and bimorph cantilever based TMEG and arrays with the resistance varying from 1 Ω to 10 MΩ.

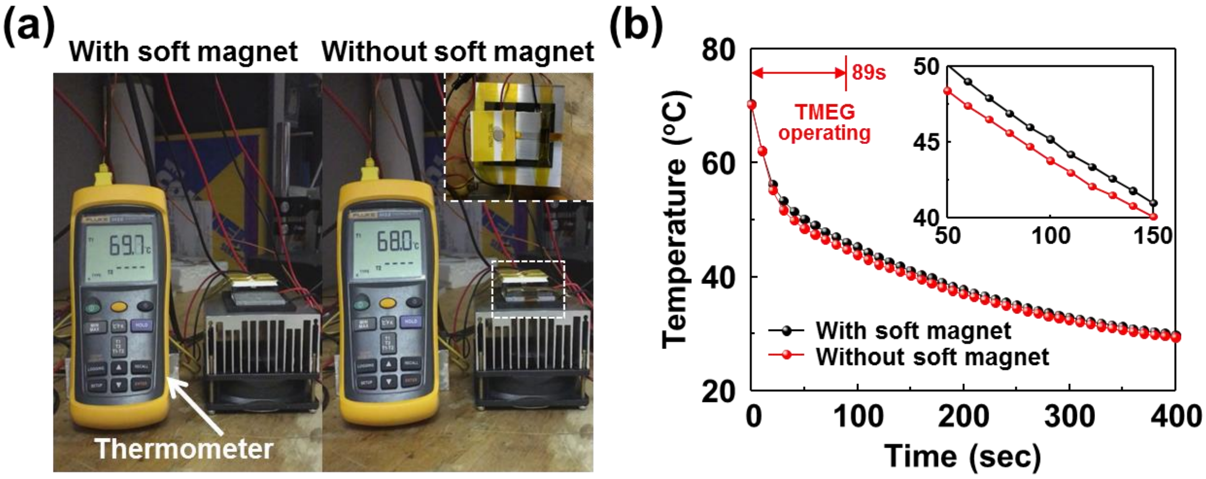

Figure S6. Heat dissipation of TMEG. (a) Snapshots of TMEG attached to Peltier heater and (b) surface temperatures on Peltier heater with TMEG and without TMEG.

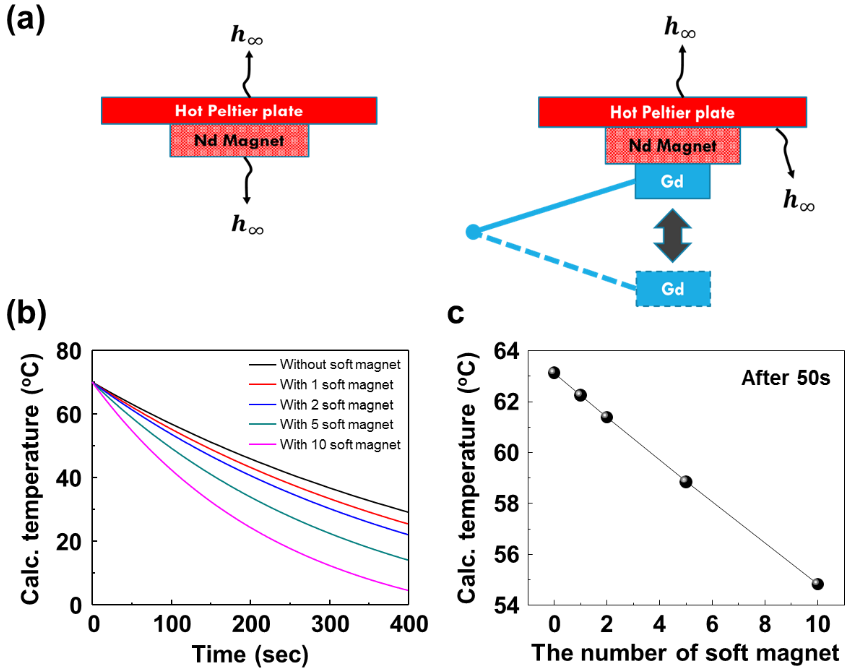

Figure S7. Analytical modeling for heat dissipation of TMEG. (a) Schematic of modeling setup for hot side with and without soft magnet. (b,c) Calculated temperature on hot side with the number of soft magnet.

Supplementary Movies

Movie 1. The video shows unimorph cantilever based TMEG operated by phase transition of soft magnet under thermal gradient of 80°C.

Movie 2. The video shows series connected three green LEDs simultaneously lighted up by TMEG arrays.

Movie 3. The video shows two commercial green LEDs driven by bimorph cantilever based TMEG mounted onto a central processing unit (CPU) inside the desktop.
